# A multidomain bio-inspired feature extraction and selection model for diabetic retinopathy severity classification: an ensemble learning approach

**DOI:** 10.1038/s41598-023-45886-7

**Published:** 2023-10-30

**Authors:** Posham Uppamma, Sweta Bhattacharya

**Affiliations:** School of Information Technology and Engineering, Vellore Institute of Technology, Vellore, Tamilnadu 632014 India

**Keywords:** Computer science, Information technology, Retinopathy of prematurity

## Abstract

Diabetes retinopathy (DR) is one of the leading causes of blindness globally. Early detection of this condition is essential for preventing patients' loss of eyesight caused by diabetes mellitus being untreated for an extended period. This paper proposes the design of an augmented bioinspired multidomain feature extraction and selection model for diabetic retinopathy severity estimation using an ensemble learning process. The proposed approach initiates by identifying DR severity levels from retinal images that segment the optical disc, macula, blood vessels, exudates, and hemorrhages using an adaptive thresholding process. Once the images are segmented, multidomain features are extracted from the retinal images, including frequency, entropy, cosine, gabor, and wavelet components. These data were fed into a novel Modified Moth Flame Optimization-based feature selection method that assisted in optimal feature selection. Finally, an ensemble model using various ML (machine learning) algorithms, which included Naive Bayes, K-Nearest Neighbours, Support Vector Machine, Multilayer Perceptron, Random Forests, and Logistic Regression were used to identify the various severity complications of DR. The experiments on different openly accessible data sources have shown that the proposed method outperformed conventional methods and achieved an Accuracy of 96.5% in identifying DR severity levels.

## Introduction

People who have diabetes are more likely to develop diabetic retinopathy (DR), which is a degenerative disease of the retina. In the next few years, it is expected to affect an increasing number of individuals, as it is now the main cause of blindness among adults who are of working age worldwide. In 2017, a significant number of people were potentially susceptible to type 2 diabetes, and the number of people who fall into this category is also steadily growing around the globe. Most of these DR patients were between 35 and 59 years old, and 1 in 2 people among the 212 million were unaware of their disease.

Numerous statistical studies conducted across the globe reveal the fact that diabetic retinopathy has acted as a significant public health issue in the present society. As per the data of 2023, the prevalence of diabetic retinopathy is estimated to be 34.6%^1^. The 74th World Health Organization (WHO) study revealed that more than 420 million people had diabetes in 2021, and 578 million were predicted to have the disease by 2030. The prevalence of diabetes is rising globally, which is further increasing the number of people who are afflicted by DR. It is also estimated that the number of DR patients worldwide is predicted to rise from 103.12 million to 160.5 million between 2020 and 2045, with 44.82 million individuals reporting visual issues. This establishes the role of DR in the potential development of a huge global, economic, and public health problem^2^. According to the International Diabetes Federation (IDF)^3^, a total of 537 million individuals in the world between the ages of 20 and 79 make up 10% of the world's population are estimated to have diabetes mellitus in 2021. The statistical data reveal that diabetes was responsible for a total worldwide health expenditure of USD 996 billion in 2021, further indicating growth of 316 percent over the course of the previous 15 years. In addition, it is also observed that in low and middle-income countries four out of every five people are diabetic patients. It is further projected that by 2030, the total number of people to likely suffer from diabetes will be 643 million which is further going to increase to 783 million by 2045^4^.

**Figure 1:**
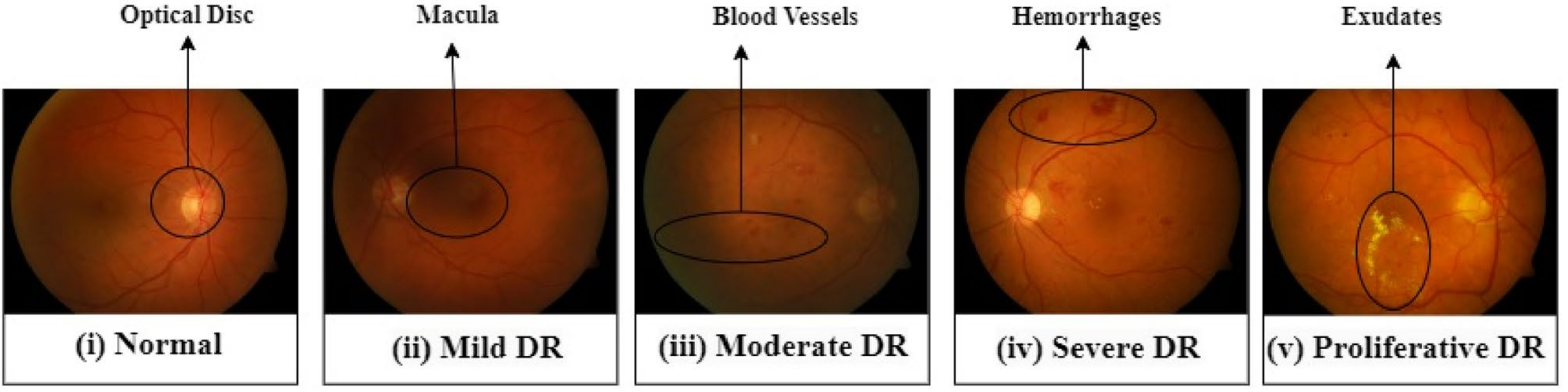
Diabetic retinopathy sample fundus images with severity grading levels. Source: diaretdb1 (standard diabetic retinopathy database).

An abnormal rise and fall in blood sugar levels are the initial symptoms of diabetes. Usually, glucose in the body is converted into energy, which lets people perform their daily tasks. But with the abnormal increase in blood sugar levels, the extra blood sugar produced has no choice but to build up in the blood vessels of various body organs such as the eye^5–7^ acting as the root cause of the disease. Ophthalmologists classified DR into three categories based on its severity level: normal, NPDR (non-proliferative diabetic retinopathy), and PDR (proliferative diabetic retinopathy). The term "NPDR" refers to the initial phase of a disease wherein there are few or no specific symptoms, while it is subsequently classified into three categories: mild level, moderate level, and severe level. The most significant symptom of NPDR is blurred eye vision, night vision problems, small regions of inflammation in the retina's blood vessels, and sometimes a few retinal veins blocked by hemorrhages, leading to reluctant blood flow in the eye. PDR is an advanced form of diabetes. In this stage, the retina lacks oxygen due to circulation issues. Hence, fragile blood vessels get affected in the retina, and vitreous, which is a gelatinous substance located in the posterior segment of the eyeball. The center of the eye (vitreous), which contains new blood vessels, eventually starts leaking blood, resulting in vision impairment^8,9^. The severity levels of DR identification using fundus images as specified in Fig. 1.

Bioinspired optimization algorithms are developed considering ideas and inspirations acquired from the natural biological evolution process to produce new and resilient competing computational approaches. These are potential options for dealing with difficult optimization challenges^10^. An example of such an approach is the Firefly algorithm^11^, which is an efficient way to select features used to perform tasks pertaining to medical image analysis. These algorithms seek to find an optimal subset of features by simulating the natural processes of attraction and repulsion and are based on natural phenomena of the behavior of the fireflies. However, the algorithm requires high computational power during the search process. The study of diabetic retinopathy is a significant field of research and is a difficult and challenging topic, although there are many optimistic improvements in the works. The researchers are using new imaging techniques that utilizes Artificial intelligence as a part of the machine learning and deep learning models to detect diabetic retinopathy in early stages with enhanced accuracy. The present study thus proposes a method for identifying DR severity levels from retinal images that begins with image pre-processing and augmentation, followed by segmenting the optical disc (OD), the macula, blood vessels, exudates, and hemorrhages through an adaptive thresholding process. After the images are segmented, the model further implements a bio-inspired feature selection process which enables the most significant features to be fed into an ensemble learning model. In this process, multidomain features are extracted from the retinal images, including frequency, entropy, cosine, gabor, and wavelet components. Then, Novel Modified Moth Flame Optimization (MMFO) is used which is based on feature selection and contributes to the maximization of feature variance levels. The model is further trained using multiple machine-learning algorithms namely Naive Bayes (NB), k Nearest Neighbours (kNN), Support Vector Machine (SVM), Logistic Regression, Multilayer Perceptron (MLP), and Random Forests as part of an ensemble model. The experiments performed on an enlarged set of data samples reveal the advantageous aspects of the ensemble learning model in comparison to the conventional approaches achieving enhanced accuracy in determining the severity levels of DR.

The distinctive contributions of the research are as follows:

- An extensive review of numerous applications of machine learning and deep learning implementations for DR detection and classification, emphasizing the contributions and related limitations.
- The implementation of image pre-processing and augmentation techniques being applied to the DR dataset from the retinal images, multidomain characteristics should be extracted.
- The process of implementing a modified moth flame algorithm of feature selection significantly cuts down on the number of features necessary for classification, thereby improving the effectiveness and interpretability of the model.
- The optimized features are classified by using machine learning algorithms NB, kNN, SVM, logistic regression, MLP, and random forests combined as an ensemble model, yielding optimized results in classifying the extent of complications of DR.

The organizational structure of this article is as follows in the Literature Review, related to the state-of-the-art work implementations using various ML and DL (deep learning) techniques applied to DR, the “Proposed methodology” section provides detailed knowledge about the proposed framework and related methodologies, and the “Result analysis” section highlighted the experimental results and comparative analysis of the proposed model, followed by the Conclusion and future scope in the area of research.

## Literature review

People who have diabetes are at an increased risk of developing DR, which is a leading cause of visual impairment. It is extremely important to identify DR at an early stage and treat it appropriately to prevent eyesight loss. Retinal fundus images, which can be evaluated using machine learning algorithms, are a method that can be utilized to determine the seriousness of DR^12,13^. This section highlights some of the models developed for evaluating DR intensity levels in this study. The authors in^14^ have proposed a synergic adversarial label learning (SALL) framework to identify relevant retinal semantic labels and feature spaces. This framework is similar to the deep convolutional neural network model^15^. However, the model used limited data sources yet yielded improved results. Similarly, the proposed generative adversarial network model^16^ was applied to higher-order fundus images for lesion segmentation wherein channel attention and multi-scale discriminator methods were used to identify large and small optimized retinal fields.

CNNs are a specific kind of DL model that has seen significant application in the field of DR severity prediction use cases. In^17^, the authors proposed a hybrid detection and classification framework to detect DR using a voting method. This framework used both image segmentation and feature selection approach to design diversified optimal feature selection to identify the DR severity classes with enhanced outcomes. However, this task was quite tedious because considering the complexity of the disease, the existence of a large amount of variability in the degree of detail of the eye retinal images, and the requirement for accurate and reliable classification performance levels via the use of UNet and Multiple Scale Attention Network (MSA-Net)^18,19^. The authors in^20^ proposed a deep graph correlation network (DGCN) that provides an innovative path for automated DR classification and other computer-assisted medical diagnosis systems. Similarly, the proposed technique utilized DenseNet169’s encoder and Convolutional Block Attention Module (CBAM)^21^ to construct a visual embedding for automated DR diagnostics. Moreover, the authors in^22^ presented a lightweight CNN model as an end-to-end DR-Net (EDR-Net) to reduce the computational cost of training large datasets. A two-stage framework^23^ for automatic DR classification was proposed, which used two different U-Net models to separate the OD (optic disc) and BV (blood vessels) in the pre-processing step. These models could automatically learn the image characteristics of the retinal fundus and further accurately categorize the images into various intensity levels. The authors in^24^ proposed a hybrid retinal image enhancement algorithm to improve image quality and further remove noise from the retinal fundus images. Moreover, the feature extraction method ResNet50 was applied to optimize the image features, yielding enhanced classification results. There have been many different iterations of CNNs developed, such as VGG, ResNet, and Inception, each of which achieved a different degree of effectiveness^25–27^.

Thus, it is quite established that transfer learning models perform well and require significantly less data for training than models that learn from inception and other use cases^28,29^. The important aspects of an image can be brought to the viewer's attention through various attention techniques. To estimate the intensity of DR, deep learning models with attention mechanisms have been developed to enhance the performance of the models^30,31^. These models have produced interesting findings, but additional investigation is required to establish the possibility of their success in identifying the characteristics^32^. Deep learning models known as capsule networks are comparatively recent developments that have demonstrated significant in the field of image categorization in real-time applications^33,34^. The proposed framework, DenseNet121, and the Oculi technique^35^ developed an optimized transfer learning model to detect DR severity levels. The framework affected the performance of the model in terms of the standardized data samples. The models are also intended to be more resistant to image transformations and aberrations than conventional CNNs because they have been developed to be more durable. The intensity of DR has been estimated using capsule networks, with studies reporting enhanced performance compared to conventional CNNs^36^.

The authors^37^ presented a detailed review of various deep and machine learning techniques, considering image enhancement techniques, to identify various complications in DR. In order to overcome issues pertaining to handling of imbalanced dataset, the authors in^38^ presented a hybrid convolutional neural network involving a grey wolf optimization framework to extract and select the optimal features yielding an optimal classification of DR. Similarly, the authors in^39^ proposed an improvised version of grey wolf optimization in association with the genetic algorithm to handle the diversified dataset population and perform optimal feature selection. These features are fed into the CNN classifier to classify the DR severity level. The authors in^40^ considered a transfer learning approach wherein a two-stage U-Net architecture was proposed for DR segmentation and classification using the CNN-SVD (singular value decomposition) model. This framework included an Inception-V3 model to extract the most relevant features which was further trained using the CNN-SVD model generating enhanced performance and ensuring reduced model complexity.

## Proposed methodology

Considering the review of the existing models that have been used to identify severity levels of diabetic retinopathy, it can be observed that these models use general-purpose deep learning classifiers, which have lower efficiency levels. The conventional models have higher complexity and cannot be applied to multi-institutional data samples. To conquer these problems, this section briefly explains the design of an augmented bio-inspired multidomain feature extraction and selection model for diabetic retinopathy severity identification using an ensemble learning process.

**Figure 2:**
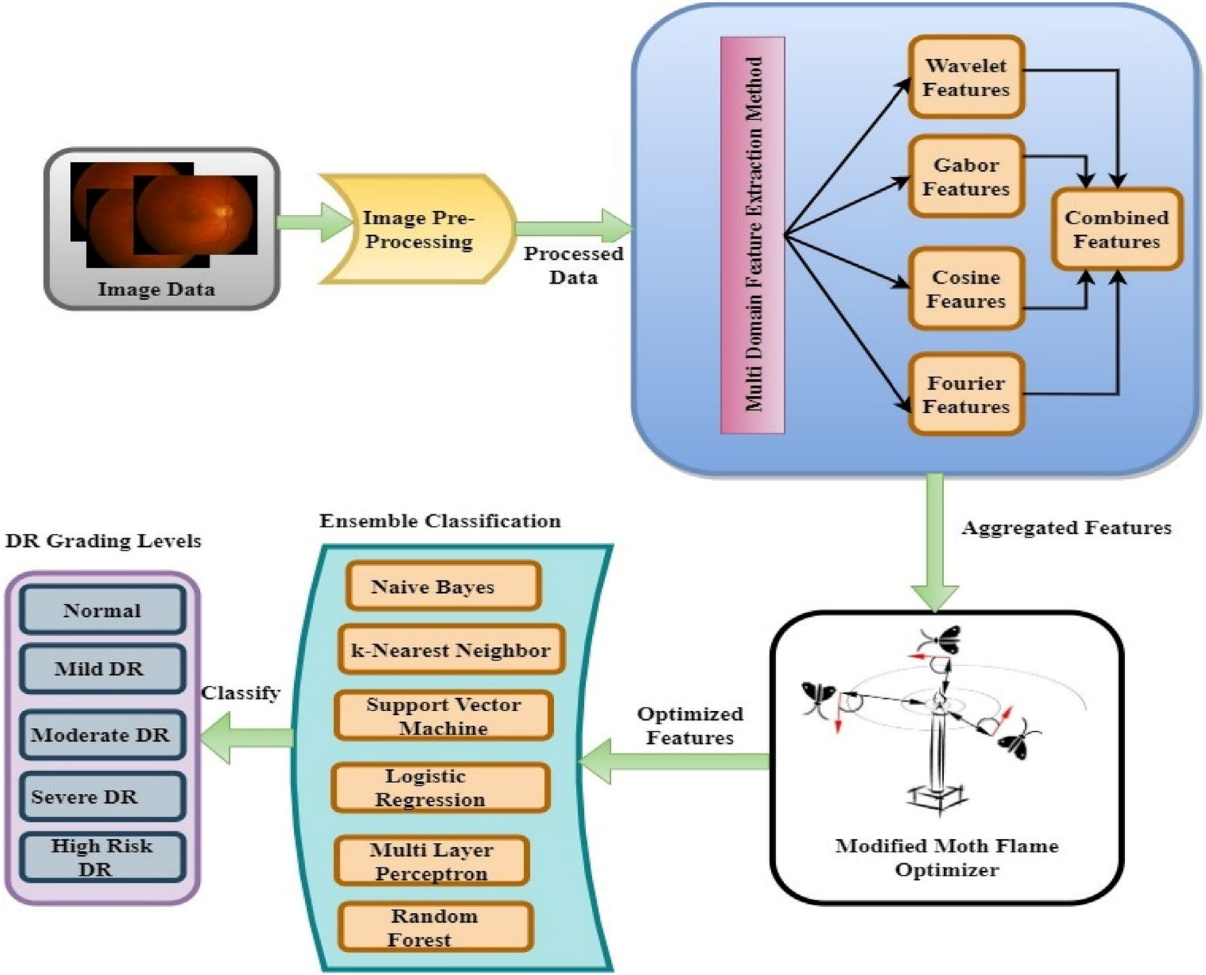
Proposed model architecture for DR classification.

The proposed approach in Fig. 2, is observed that in order to identify DR severity levels from retinal images, it is initially necessary to segment the optical disc (OD), macula, blood vessels, exudates, and hemorrhages using an adaptive thresholding process^41^. Once the images are segmented, the model uses bioinspired feature selection and ensemble learning, wherein multidomain features^42^ from the retinal images, including frequency, entropy, cosine, gabor, and wavelet components are extracted. These data features are fed into a novel Modified Moth Flame Optimization (MMFO)^43^ feature selection model that assists in maximizing feature variance levels and identifying optimized features. These optimized features are trained using an ensemble model that includes various ML algorithms such as Naive Bayes (NB)^44^, k Nearest Neighbours (kNN)^45^, Support Vector Machine (SVM)^46^, Logistic Regression^47^, Multilayer Perceptron (MLP)^48^, and Random Forests^49^. The ensemble model combines the results of many different models to increase accuracy and robustness.

### Dataset description

In this framework, two similar datasets are used, namely the Indian Diabetic Retinopathy Image Dataset (IDRiD)^50^ and the standard diabetic retinopathy database (DIARETDB1)^51^. The IDRiD dataset has 516 authentic fundus images which are isolated from the ratio of training (413) and testing (103). This dataset contains images annotated with disease severity at the pixel level of diabetic retinal lesions. The DIARETDB1 dataset has 89 authentic color fundus images consisting of 5 healthy sign images and the remaining 84 having DR-related symptoms. Both the dataset images were captured using a 50-degree FOV (field of view) fundus camera. These datasets have grades ranging from 0 to 4 as healthy, mild, moderate, severe, and proliferative to classify DR severity grading levels. Table 1 provides a detailed description of the datasets.

**Table 1:** A detailed description of the Dataset.

DR stages	IDRiD dataset	DIAREDTDB1 dataset	Disease detail
No DR	100	5	Healthy retina
Mild DR	153	17	Microaneurysms
Moderate DR	103	12	Few Microaneurysms
Severe DR	100	16	Intraretinal microvascular abnormalities, Hemorrhages
Proliferative DR	60	27	Exudates

### Image augmentation and preprocessing

The proposed model initially collects retinal images of different diabetic and non-diabetic patients. The image augmentation technique is used to increase the number of data samples to fit the training model efficiently. This technique is mainly applied to increase the number of image samples using rotation, zoom, and shear functions. During the training of the model, the use of these parameters produces images with the aforementioned characteristics. The images were collected for grade 0 (normal person), grade 1 (mild DR), grade 2 (moderate DR), grade 3 (high DR), and grade 4 (very high DR) cases. The collected images are then converted into the optical disc (OD), macula, blood vessels, exudates, and hemorrhages using multidomain feature extraction^42^ and segmented through an adaptive thresholding process.

This adaptive thresholding process^41^ is used for image segmentation wherein a small set of nearest pixel intensities for a specific time is observed. This method is specifically used to decrease computational time consumption. It computes the threshold value (th) for every specific neighborhood region to perform the image segmentation process. In comparison to the global thresholding value, adaptive thresholding enables the segmentation process to become robust providing enhanced contrast and brightness to the fundus images. It acts as a powerful segmentation tool for segmenting the fundus image features accurately and effectively. Also, the process can easily be implemented on a wide variety of fundus image features encouraging researchers to use the same. However, there exist associated challenges pertaining to noise generation while segmentation of tiny image features. Adaptive thresholding is thus considered to be an extremely popular technique in image processing to segment images and extract specific regions of interest. The process of adaptive thresholding is elucidated as follows:

*Optical disc (OD)* The optical disc, also known as the optic nerve head is the exit point for the ganglion cell axons that leave the eye. Since there does not exist any rods or cones overlying the optic disc, it can be identified as a small blind spot in each of the eyes. OD segmentation is usually based on shape and brightness. Equation (1) is used to extract OD as follows

1 $$OD = {b}_{in}\left(I, th\left(I, 0.4,FP,dark\right)\right)$$

where, ‘b_in_’ and ‘th’ are binarization and thresholding operations, while ‘FP’ represents the foreground polarity of the image sets, ‘I’ represents the input grayscale image, the threshold value of 0.4, and the 'dark' polarity is chosen through the empirical analysis.

*Macula* Macula is a region of high intensity in the central part of the retina and is estimated as follows

2 $$Macula= {b}_{in}\left(I, th\left(I, 0.6,FP,bright\right)\right)$$

where the threshold value of 0.6 and ‘bright’ polarity are chosen through the empirical analysis.

*Blood Vessels* Blood vessels are thin, elongated structures with varying intensities, and are follows

3 $$BV = {b}_{in}\left(I,th\left(I, 0.5,DP,right\right)\right)$$

4 $$BV = close\left(BV, structure\left(disc, 5\right)\right)$$

where the threshold value of 0.5 and ‘bright’ polarity are chosen empirically, and a closing operation in morphology that uses a size-structuring element in the form of a disc is 5 selected through the empirical analysis.

*Exudates* Exudates are yellowish-white spots with high intensity and irregular shapes, which are identified as follows

5 $$Exudates ={b}_{in}\left(I, th\left(I, 0.3, FP, bright\right)\right)$$

6 $$Exudates = open\left(Exudates, structure\left( disc,10\right)\right)$$

where in the threshold value of 0.3 and bright polarity are chosen empirically, and a closing operation in morphology that uses a size-structuring element in the form of a disc is 10 for different image sets.

*Hemorrhages* Hemorrhages are bright red or dark spots with irregular shapes which are evaluated as follows

7 $$Hemorrhages = {b}_{in}\left(I, th\left(I, 0.7,FP,bright\right)\right)$$

8 $$Hemorrhages = open\left(Hemorrhages, structure\left(disc, 3\right)\right)$$

where, the threshold value is 0.7 and bright polarity is chosen empirically. The closing operation in morphology using a size-structuring element in the form of a disc is 3 as considered in the case of different image sets.

These images are represented in multiple domains by estimating frequency, entropy, spatial, approximate, and detailed features. The frequency features are evaluated using Discrete Fourier Transform (DFT), wherein ‘N_q_’ is the number of input image pixels (m) used for the feature extraction process.

9 $$DF{T}_{m}=\sum_{n=1}^{{N}_{q}}{p}_{n}*\left[\mathit{cos}\left(\frac{2*\pi *m*n}{{N}_{q}}\right)-\sqrt{-1}*\mathit{sin}\left(\frac{2*\pi *m*n}{{N}_{q}}\right)\right]$$

Similarly, the entropy features are estimated using Discrete Cosine Transform (DCT) as follows

10 $$DC{T}_{i}=\frac{1}{\sqrt{2*{N}_{q}}}*{p}_{n}\sum_{m=1}^{{N}_{q}}{x}_{i}*\mathrm{cos}\left[\frac{\sqrt{-1}*\left(2*m+1\right)*\pi }{2*{N}_{q}}\right]$$

The spatial features are calculated using Gabor analysis as follows

11 $$G{\left(p,s\right)}_{r}={e}^{\frac{-x{`}^{2}+{\partial }^{2}*{y}^{{\prime}2}}{2*{\varnothing }^{2}}}*\mathrm{cos}\left(2*\frac{pi}{\lambda }*{{p}^{\prime}}\right)$$

where, p and s are indices and values of features, while ∂, $\varnothing$, and $\lambda$ represent constants for angular and wavelength augmentation operations. In combination with these features, approximate and detailed wavelet components were also calculated as follows

12 $${W}_{j}=\frac{{p}_{n}+{p}_{m+1}}{2}$$

13 $${W}_{k}=\frac{{p}_{n}-{p}_{m+1}}{2}$$

All these feature vectors are combined to form the Diabetic Retinopathy Feature Vector (DRFV), which could include redundancies that are reduced via the use of a feature selection approach.

### Modified moth flame optimization (MMFO) algorithm

Moth Flame Optimization is a type of swarm intelligence-inspired algorithm. The algorithm solves numerous authentic problems in various provinces and was developed by Sayyad Mirjalili^43^. It represents a population-based mechanism to specify the navigation behavior of moths in nature. The artificial moth imitates the navigation technique used by actual moths, which is referred to as transverse orientation. This is accomplished by maintaining a stable angle with a distant source of flame in order to orient itself. This mechanism is initially generated randomly in solution space, determining each moth’s fitness function and setting the best position for the moth. Based on the spiral behavior mechanism, moths' positions are changed concerning the flame and updated to new positions. The bioinspired feature selection method significantly reduces the number of features required for classification, improving the interpretability and effectiveness of the model sets. The proposed Modified Moth Flame Optimization (MMFO) is observed to be robust and simplified while performing hyperparameter optimization for optimal feature selection wherein maximum class-level feature variance is considered. The traditional MFO, although has multiple advantages being simple, flexible, and validated across various applications has challenges of being constrained in generating local optimal solutions providing inferior convergence rates. The MMFO algorithm provides the ability to include maximum variance in the class-level features to achieve optimal feature selection. Also, it enhances the exploration capability of the MMFO algorithm in terms of minimizing the number of features and keeps a smaller selection of relevant features without impairing the system's performance and convergence cost. In this regard, MMFO-measured minimal fitness values are extracted from the dataset considering the diabetic retinopathy feature vectors (DRFV). The process is iterated until the optimal feature selection is achieved. Although the algorithm proves to be efficient as part of various ML and DL frameworks for optimal feature set selection but is found to be computationally expensive while handling larger volumes of dataset.

The algorithm is based on the characteristic of moths which by nature fly in various directions, namely one-dimensional, two-dimensional, and three-dimensional views. These can be represented as follows

14 $${M}_{th}=\left[\begin{array}{ccc}{mth}_{\mathrm{1,1}}& {mth}_{\mathrm{1,2}}\dots \dots & {mth}_{1,d}\\ \vdots & \dots & \vdots \\ {mth}_{x,1}& {mth}_{x,2}\cdots & {mth}_{x,y}\end{array}\right]$$

Here x specifies the number of moths, and y specifies the dimensions of each moth in the solution area. These can be utilized to calculate the fitness function vectors represented as follows

15 $${f}_{tn}=\left[\begin{array}{ccc}{ftn}_{\mathrm{1,1}}& {ftn}_{\mathrm{1,2}}\dots \dots & {ftn}_{1,d}\\ \vdots & \dots & \vdots \\ {ftn}_{x,1}& {ftn}_{x,2}\cdots & {ftn}_{x,y}\end{array}\right]$$

Here ‘f_tn_’ specifies the fitness value vector for each solution space. The position of the moths gets updated to calculate the global optimum values to solve the optimization problems as follows

16 $$\mathrm{MMFO }= \left(\mathrm{L},\mathrm{M},\mathrm{P}\right)$$

17 $${M}_{th} = (\mathrm{up}(\mathrm{m})-\mathrm{lw}(\mathrm{n}))*\mathrm{ random}()+\mathrm{lw}(\mathrm{m}))$$

18 $$\mathrm{Flmno }=\mathrm{ round}(\mathrm{X}-1 *\mathrm{X}-1/\mathrm{M}))$$

Here L, M, and P represent the moth’s random location, the movements of the moths in the search area, and finishing the search process, respectively. Where up and lw refer to upper bound and lower bound values to calculate the random distribution function. To achieve the best optimal values in the solution search space by updating the positions of the moths. Where M and X represent the number of iterations and flames at the maximum level respectively.

**Algorithm 1:**
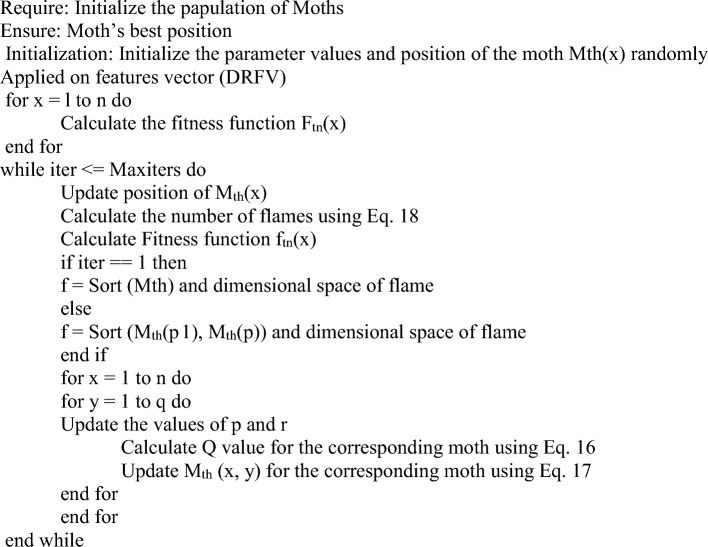
The pseudo-code for the Modified Moth Flame Optimization Algorithm.

Initially, a set of NM moths are generated by the selection of N features specified as follows

19 $$N=STOCH\left(LM*N\left(DRFV\right), N\left(DRFV\right)\right)$$

where LM is the moth Learning Rate, N(DRFV) represents several extracted multidomain features, and STOCH represents a stochastic number generation process. For each of these selected features, evaluate moth flame fitness (fm) as follows

20 $$the \, {f}_{m}=\sqrt{\frac{\sum_{i=1}^{N}{\left({x}_{i}-\sum_{j=1}^{N}\frac{{x}_{j}}{N}\right)}^{2}}{N}}$$

Similarly, generate NM different moths, and find the fitness threshold as follows

21 $${f}_{th}=\frac{1}{NM}\sum_{i=1}^{NM}f{m}_{i}*LM$$

22 $${f}_{New}={f}_{Old}\bigcup_{i=1}^{fm>{f}_{th}}STOCH\left(f\left(i\right)\right)$$

moths with f_m_ < f_th_ are discarded, and their features are updated using Eq. (22). Where ‘f’ represents features of the current set of moths and the movement of the other moths is directly passed to the next set of iterations. This process is repeated for ‘NI’ iterations, and their fitness and internal configurations are modified in each set of iterations. Once all iterations are completed, the initial feature sets are estimated

23 $${f}_{Initial}=\bigcup_{i=1}^{fm>{f}_{th}}f\left(i\right)$$

These features are further processed using other iterations of Moth Flame Optimization (MFO) and a set of NM moths gets generated again by the selection of N features from the f(Initial) sets through Eq. (23).

24 $${N}_{New}=STOCH\left(LM*N\left({f}_{Initial}\right), N\left({f}_{Initial}\right)\right)$$

For each of these selected features, inter-class moth flame fitness is evaluated f_m_(in) as follows

25 $${f}_{m}(in)=\sqrt{\frac{{\sum_{a=1}^{m}{(x}_{a}-\frac{\sum_{i=1}^{m}\sqrt{\frac{\sum_{j=1}^{n}{{(x}_{j}-\frac{\sum_{k=1}^{n}{x}_{k}}{n})}^{2}}{n-1}}}{m})}^{2}}{m-1}}$$

where, m represents features of one class, while ‘m’ represents features of other classes. Similarly, generate ‘NM’ different moths and the fitness threshold is determined as follows

26 $${f}_{th}=\frac{1}{NM}\sum_{i=1}^{NM}{f}_{m}{(in)}_{i}*LM$$

27 $${f}_{New}={f}_{Old}\bigcup_{i=1}^{{f}_{m}(in)>{f}_{th}}STOCH\left(f\left(i\right)\right)$$

Moths with ${f}_{m}\left(in\right)<{f}_{th}$ are discarded, and their features are updated through Eq. (27). Where f represents features of the current set of moths and the movement of other moths is directly passed to the next set of iterations. This procedure is performed until ‘NI’ iterations are completed, and the relevant fitness and internal configurations are modified in each set of iterations. Once all iterations are completed, the final feature sets are estimated as follows

28 $${f}_{final}=\bigcup_{i=1}^{{f}_{m}(in)>{f}_{th}}f\left(i\right)$$

All these features are fed into a set of classifiers including Naive Bayes (NB), k Nearest Neighbours (kNN), Support Vector Machine (SVM), Logistic Regression (LR), Multilayer Perceptron (MLP), and Random Forests which are combined using boosting operations.

### Ensemble learning

#### Naive bayes (NB)

The Naive Bayes classifier^44^ is a supervised machine learning method to classify training data members that are independent of each other’s specific occurrences. It is one of the most efficient algorithms that produce faster outcomes. This classifier depends on the Bayes theorem to find the probability of the conditional hypothesis.

#### kNN (k-nearest neighbours)

The kNN^45^ is a commonly used and simple form of classification in machine learning algorithms. This classifier defines the new data points by considering the nearest data points to calculate the equal value of the earlier data.

#### Support vector machine (SVM)

The SVM^46^ is a supervised learning algorithm, one of the most implemented algorithms to solve regression and classification problems. This classifier generated the extreme boundary values to create hyperplanes for the best solutions. Suppose that the dataset has two different data features and is labelled. These data features are categorized based on labelling to draw a baseline between them and classify them easily.

#### Logistic regression (LR)

Logistic Regression (LR)^47^ is among the simplest ML techniques, and it can be used to forecast continuous variables. This algorithm specifies the relationship between dependent and independent values and determines its effect on the resultant dependent variable. The objective is to calculate the best prediction in order to decrease the error rate between the dependent and independent values. The mean square error cost function average is calculated to minimize the error rate.

#### Multilayer perceptron (MLP)

A multi-layer perceptron^48^ contains Input, output, and hidden layers the three types of layers, and is generally called a feed-forward neural network. These input layers are combined to send the information to each hidden layer in the network, and these hidden layers generate one single output. This algorithm mainly uses the back-propagation technique to train the model as well as image detection and analysis.

#### Random forests (RF)

The Random Forest algorithm^49^ helps to solve regression and classification problems on the basis of ensemble learning. This algorithm depends on the various decision trees and takes the average of the given data to improve the classification performance. Random forests use two approaches to ensure that no two trees’ behaviours are too similar: bagging and feature randomness. This causes significant heterogeneity among the trees in the model, resulting in decreased correlation among trees and greater diversity.

The final ensemble boosting method is as follows

29 $$\begin{aligned} C_{out} & = C\left( {NB} \right)*A\left( {NB} \right) + C\left( {kNN} \right)*A\left( {kNN} \right) + C\left( {LR} \right)*A\left( {LR} \right) \\ & \quad + C\left( {SVM} \right)*A\left( {SVM} \right) + C\left( {MLP} \right)*A\left( {MLP} \right) \\ \end{aligned}$$

where A and C represent the testing accuracy and output class for the given classifier, while *C*_*out*_ is the final output of the ensemble classification process. The use of ensemble classification enables improvement in classification accuracy at different DR grade levels. This accuracy, in association with metrics namely precision, recall, and delay are needed for classification. It is evaluated for different dataset samples and compared with the existing models.

## Result analysis

The proposed framework for identifying DR severity levels from retinal images is described in this section. At the outset, the retinal image classes, namely optical disc (OD), macula, blood vessels, exudates, and hemorrhages, are segmented using an adaptive thresholding process. After segmenting the images, the multidomain features namely frequency, entropy, cosine, gabor, and wavelet components are extracted from the retinal images. To maximize the feature variance and optimized feature selection, a novel modified moth flame optimization (MMFO)-based feature selection method is employed. The optimized features are trained using multiple machine-learning algorithms combined as an ensemble classification mechanism to classify the DR severity levels. These algorithms include Naive Bayes (NB), k Nearest Neighbours (kNN), Support Vector Machine (SVM), Logistic Regression (LR), Multilayer Perceptron (MLP), and Random Forests. The integrated results generated from the various models justify the superiority of the ensemble model in terms of its enhanced reliability and precision.

The proposed framework is implemented on the Windows 11 operating system with an i7 processor, 16 GB of RAM, 1 TB of SSD memory, and 4 GB of NVIDIA GPU resources using NumPy and TensorFlow libraries in a Python environment. The proposed framework has yielded superior results when compared to the existing models, namely, the Mixed Model-DR Ensemble model^17^, the Deep U-Net architecture^18^, the DGCN model^20^, and the Hybrid Retinal-DL model^24^. The performance metrics that are used to calculate the performance of the training and testing data are Accuracy, Precision, Recall and F1-Score based on the true positive (*T*_*p*_) rate, true negative (*T*_*n*_) rate, false positive (*F*_*p*_) rate, and false negative (*F*_*n*_) rates of the classes. The equations of the considered metrics are presented in Eqs. (30)–(33).

30 $$Acc\left(Accuracy\right)=\frac{{T}_{p}+{T}_{n}}{{T}_{p}+{T}_{n}+{F}_{p}+{F}_{n}}$$

31 $$Pr\left(Precision\right)=\frac{{T}_{p}}{{T}_{p}+{F}_{p}}$$

32 $$R\left(Recall\right)=\frac{{T}_{p}}{{T}_{p}+{F}_{n}}$$

33 $$F1-Score=\frac{(2*Pr*R)}{(Pr+R)}$$

### Experimental analysis

The proposed framework analyses the various DR grade classes with high degrees of precision as it uses high-density feature extraction models and MMFO integrated with an ensemble learning framework. The proposed framework yielded prominent results in comparison to the conventional DR detection and classification models, namely, the Mixed Model-DR Ensemble model^17^, the Deep U-Net architecture^18^, the DGCN model^20^, and the Hybrid Retinal-DL model^24^. Empirical studies reveal the fact best outcomes are achieved when 20–30% of the data are used for testing and 70–80% of the data are used for the purpose of training. Similarly, in case of machine learning algorithms, adjustment of the training—testing data ratio have significant influence on the performance of the model. Considering the same rule of thumb and to reduce chances of overfitting in the present study 80% of the data is used for training and the remaining 20% is used for the purpose of testing the model.

**Figure 3:**
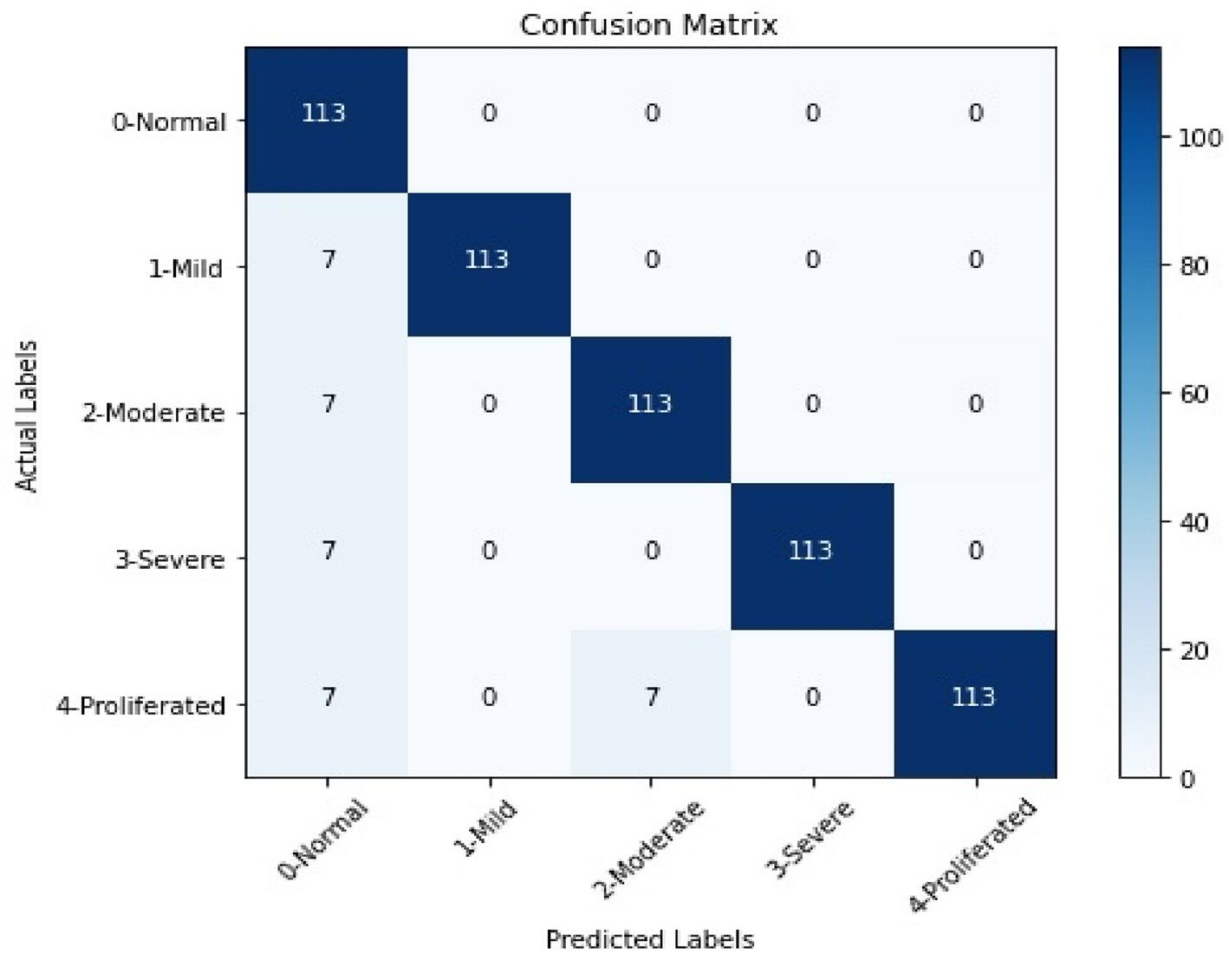
A heatmap that represents the confusion matrix of the ensemble learning model for predicting DR severity levels.

The confusion matrix is generated from the ensemble classification model considering varying severity levels (0-Normal, 1-Mild, 2-Moderate, 3-Severe, 4-Proliferated). In this regard, Fig. 3 represents the heatmap as part of confusion matrix wherein the data points are divided into actual and predicted severity levels. The heatmap highlights the highest achieved accuracy of 96% for all the severity levels.

**Figure 4:**
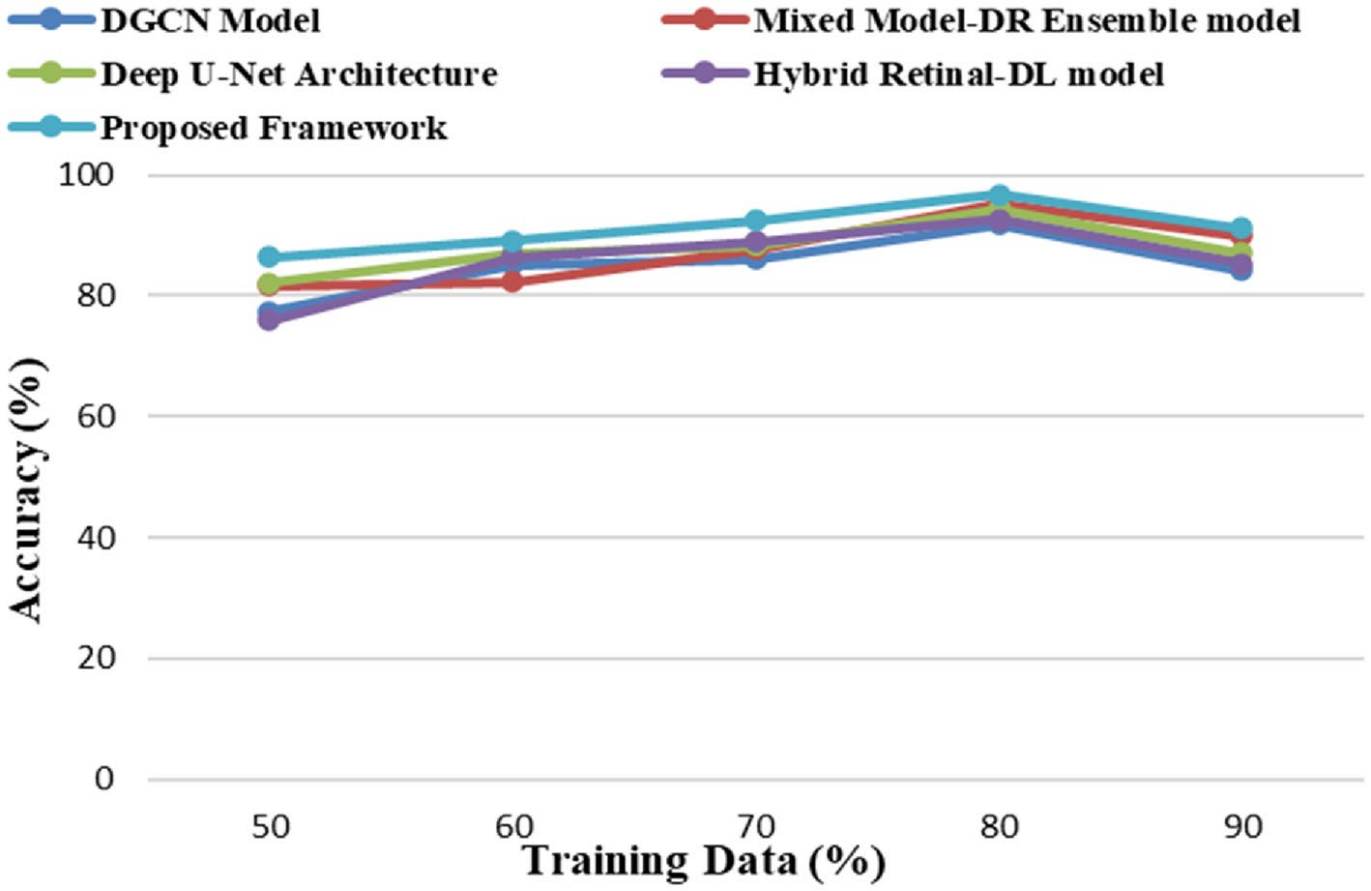
The Accuracy of severity identification for different models.

The model is further evaluated by varying the ratio of training and testing data and the resultant accuracy is generated. It is observed that the best accuracy is achieved when the training—testing data is of 80:20 ratio. The comparative analysis of generated accuracy measure of all the models are illustrated in Fig. 4 wherein it is observed that all the existing models generated enhanced accuracy considering 80:20 training—testing data ratio. More so, the proposed framework further generated superior accuracy in comparison to the other state-of-the-art models.

**Figure 5:**
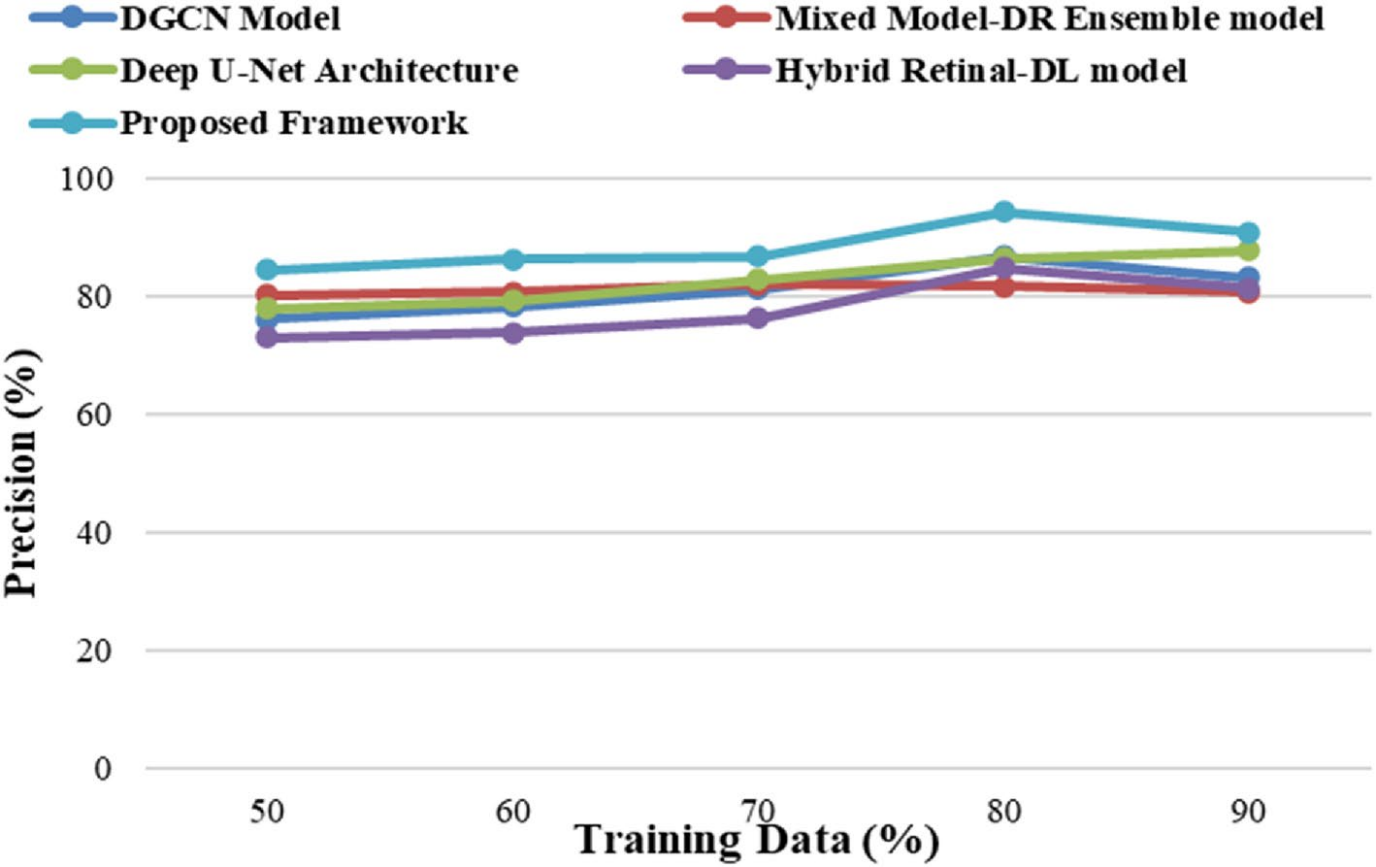
The Precision of severity identification for different models.

Due to the utilization of high-density feature extraction models and MMFO with ensemble learning, the proposed model evaluated various DR grade types with high levels of precision. The proposed model improved the DR grade classification precision value in comparison to the conventional models, namely, the Mixed Model-DR Ensemble model^17^, the Deep U-Net architecture^18^, the DGCN model^20^, and the Hybrid Retinal-DL model^24^, by varying the training data, as shown in Fig. 5. The precision was also enhanced by the application of Gabor and wavelet analysis, which aided in enhancing classification performance even with smaller data samples.

**Figure 6:**
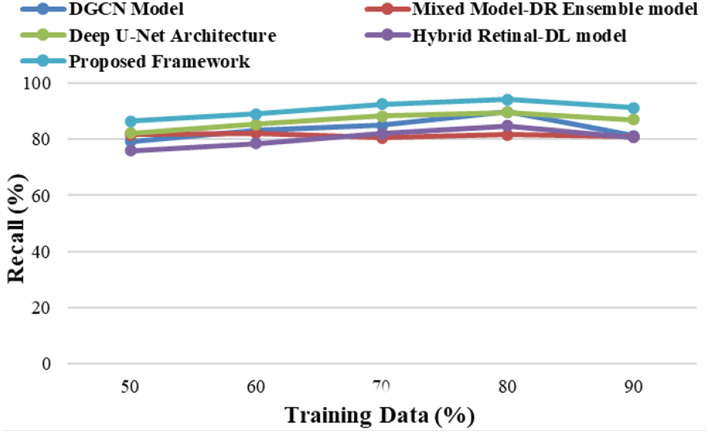
Recall of severity identification for different models.

Figure 6 reveals the fact that the proposed model yields enhanced recall measure in comparison to the conventional models, namely, the Mixed Model-DR Ensemble model^17^, the Deep U-Net architecture^18^, the DGCN model^20^, and the Hybrid Retinal-DL model^24^. The recall value achieved is also superior when measured considering varying training and testing data ratios.

**Figure 7:**
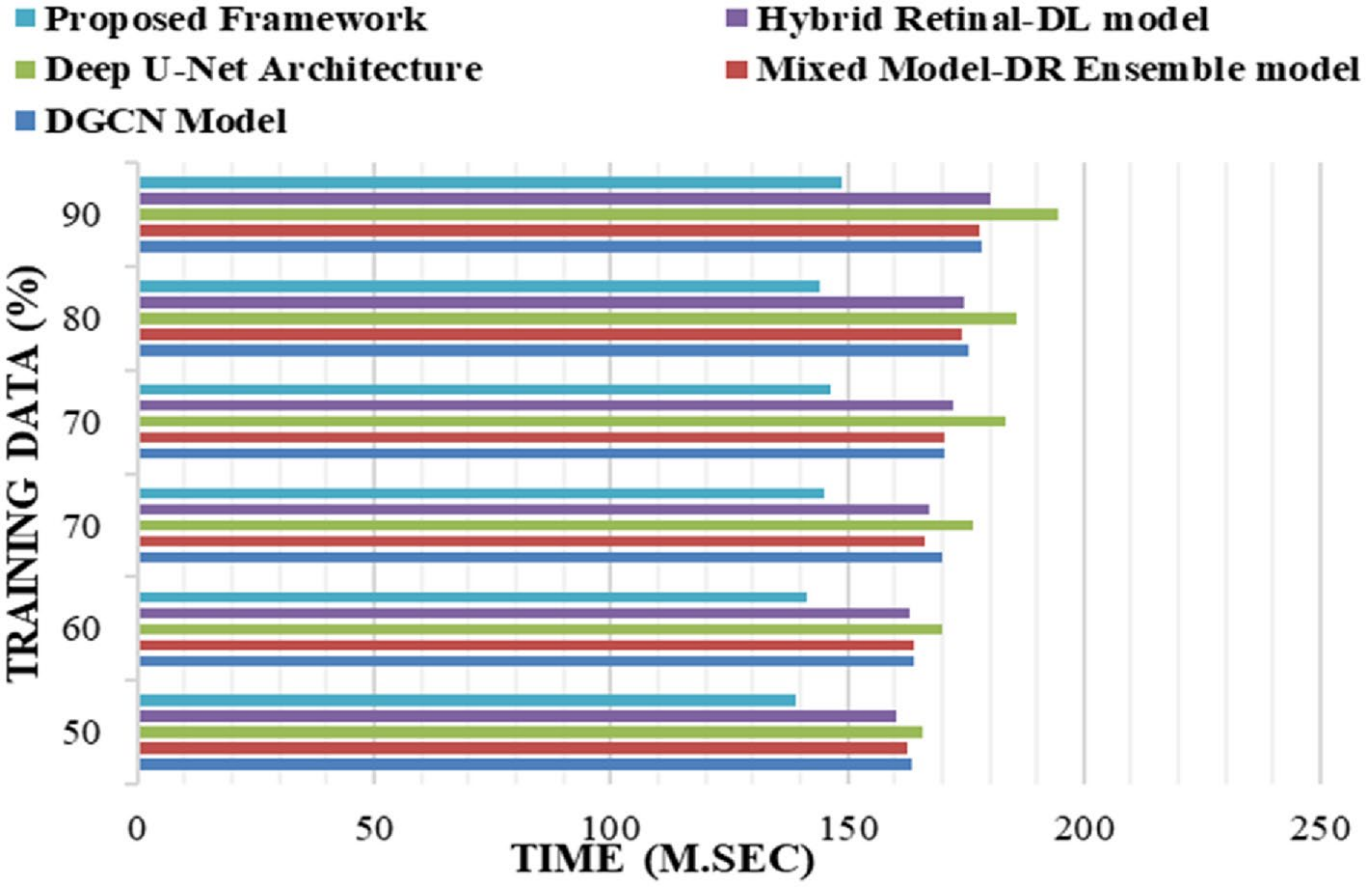
The Computational time for Training the Model in comparison to the existing models.

Figure 7 shows the computational time utilized in the training of the model wherein lower computational time is observed, which also justifies its enhanced performance in comparison to the conventional models, namely, the Mixed Model-DR Ensemble model^17^, the Deep U-Net architecture^18^, the DGCN model^20^, and the Hybrid Retinal-DL model^24^.

### Performance analysis

**Figure 8:**
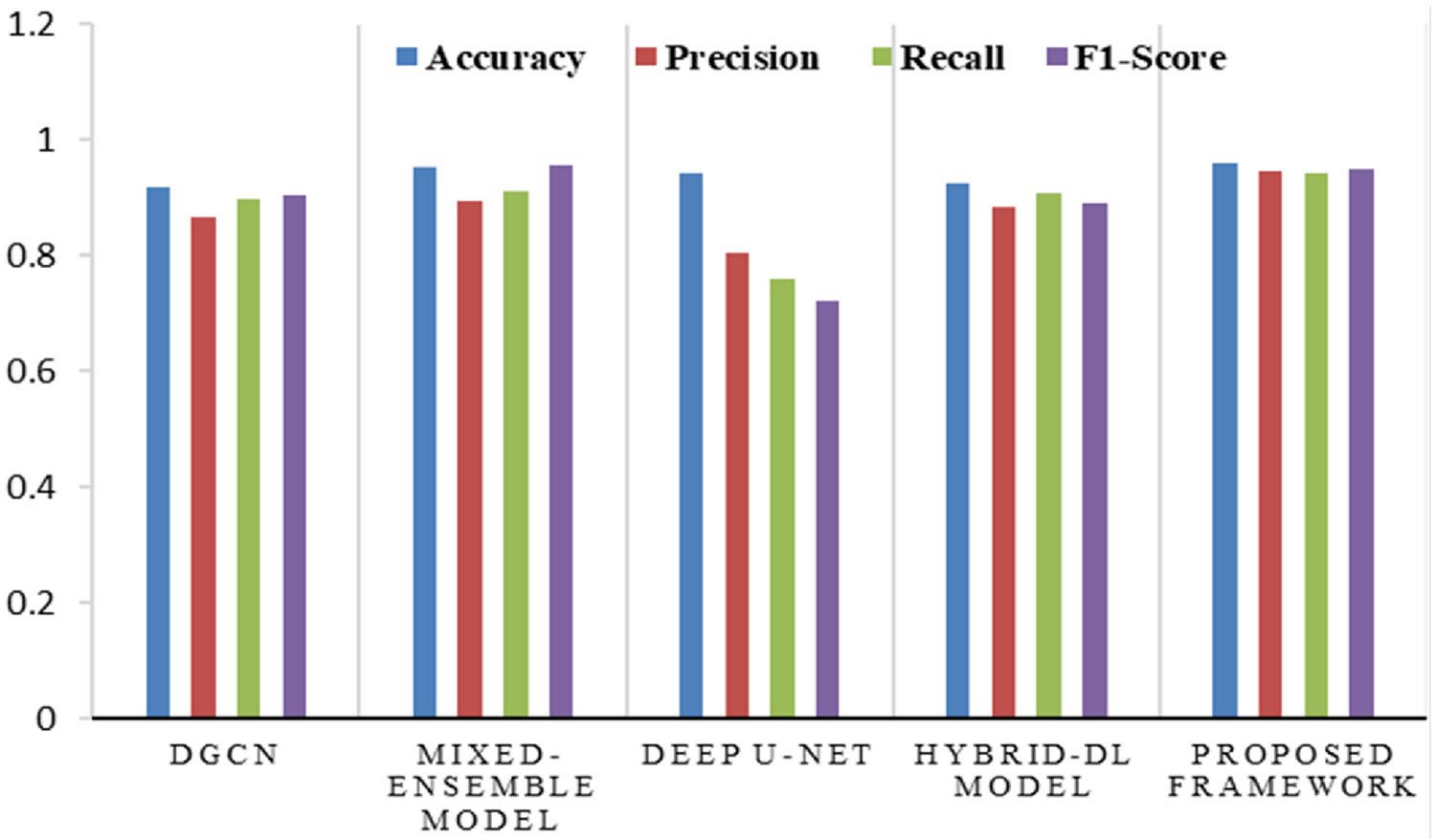
Evaluation of performance measures of the proposed framework with other conventional models.

The research community has been immensely focused on studying aspects pertaining to automatic DR classification, especially using advanced ML and DL techniques. The proposed framework in this study has yielded superior performance in comparison to the Mixed Model-DR Ensemble model^17^, the Deep U-Net architecture^18^, the DGCN model^20^, and the Hybrid Retinal-DL model^24^, and conventional models considering the aforementioned metrics. The comparative results of the performance of Mixed Model-DR Ensemble model, Deep U-Net architecture, DGCN model, and Hybrid Retinal-DL model in contrast to the proposed model considering Accuracy, Precision, Recall and F1-Score are presented in Fig. 8. Moreover, the proposed framework was evaluated considering computational time and Accuracy measures and further evaluated against the conventional models for DR classification. Table 2 represents such evaluation measures of Precision, Recall, F1-Score, and Accuracy that enabled the comparative analysis of the conventional models with to the proposed model.

**Table 2:** The performance analysis of evaluation metrics of various models.

Metrics	DGCN model	Mixed ensemble model	Deep U-Net architecture	Hybrid DL model	Proposed framework
Accuracy	0.9178	0.9539	0.9431	0.9260	0.9648
Precision	0.8683	0.8952	0.8062	0.8854	0.9474
Recall	0.8967	0.9106	0.7588	0.9067	0.9419
F1-Score	0.9053	0.9563	0.7210	0.8923	0.9490

## Conclusion

The proposed framework for identifying DR severity levels from retinal images includes an image pre-processing technique followed by the optical disc (OD), macula, blood vessels, exudates, and hemorrhages, which are segmented by utilizing an adaptive thresholding procedure. After segmenting the images, an ensemble learning model involving bio-inspired feature selection is used to extract multidomain features namely frequency, entropy, cosine, gabor, and wavelet components from the retinal images. A novel modified moth flame optimization (MMFO)-based feature selection method is employed to maximize the feature variance for achieving optimal feature selection. These optimized features are trained using various machine-learning algorithms employed during the ensemble model process. The algorithms included are Naive Bayes, k-Nearest Neighbors, support vector machine, logistic regression, multilayer perceptron’s, and random forests. The aggregated combination of the results generated from the multiple models propels the ensemble model to generate results with increased levels of reliability and precision. Due to the use of high-density feature extraction models and MMFO with ensemble learning, the proposed model further evaluates various DR-grade classes with high levels of accuracy. The proposed model is further evaluated considering computational time and accuracy metrics compared against the conventional models for DR classification by varying the training data. It is observed that the application of low-complexity feature analysis aided in accelerating the process and helped to achieve improved categorization success despite the constraint of using lesser data. The study directs the potential outcomes of the proposed approach in improving clinical decision-making and management of DR. The same framework can also be applied to predictions involving medical image analysis in other healthcare domains as well. The results generated from the model yield enhanced Accuracy, Precision, Recall and F1-Score justifying the superiority of the model in predicting diabetic retinopathy. But in practicality, often healthcare providers fail to rely on the generated predictions of the model as the output does not map with the input attributes fed in to the model failing to establish the contribution of the attributes leading to the prediction results. It is almost like a “black box” framework wherein optimized output is getting generated from the input, but users fail to get clarity on how the input impact on the resultant output. Hence, as part of the future research work, explainable AI can be applied additionally to the framework to achieve enhanced transparency and explainability to the generated prediction leading to confident decision-making in real-time healthcare scenarios.

## Data Availability

In this proposed work, two similar features of datasets are taken from different data sources available in the IEEE data port and the other is standard diabetic retinopathy database repositories, [https://ieee-dataport.org/open-access/indian-diabetic-retinopathy-image-datasetidrid], [https://www.kaggle.com/datasets/nguyenhung1903/diaretdb1-standard-diabetic-retinopathy-database] accessed on December 2022.

## References

[1] Zegeye AF, Temachu YZ, Mekonnen CK (2023). Prevalence and factors associated with Diabetes retinopathy among type 2 diabetic patients at Northwest Amhara Comprehensive Specialized Hospitals, Northwest Ethiopia 2021. BMC Ophthalmol..

[2] Xiao H, Tang J, Zhang F, Liu L, Zhou J, Chen M, Li M, Xiaoxiao Wu, Nie Y, Duan J (2023). Global trends and performances in diabetic retinopathy studies: A bibliometric analysis. Front. Public Health.

[3] Sun H, Saeedi P, Karuranga S, Pinkepank M, Ogurtsova K, Duncan BB, Stein C (2022). IDF Diabetes Atlas: Global, regional and country-level diabetes prevalence estimates for 2021 and projections for 2045. Diabetes Res. Clin. Pract..

[4] Gadekallu TR, Khare N, Bhattacharya S, Singh S, Maddikunta PKR, Ra I-H, Alazab M (2020). Early detection of diabetic retinopathy using PCA-firefly based deep learning model. Electronics.

[5] Kalyani G, Janakiramaiah B, Karuna A, Narasimha Prasad LV (2021). Diabetic retinopathy detection and classification using capsule networks. Complex Intell. Syst..

[6] Alahmadi MD (2022). Texture attention network for diabetic retinopathy classification. IEEE Access.

[7] Kaushik H, Singh D, Kaur M, Alshazly H, Zaguia A, Hamam H (2021). Diabetic retinopathy diagnosis from fundus images using stacked generalization of deep models. IEEE Access.

[8] Ouyang J, Liu S, Peng H, Garg H, Thanh DNH (2023). LEA U-Net: a U-Net-based deep learning framework with local feature enhancement and attention for retinal vessel segmentation. Complex Intell. Syst..

[9] Guo Y, Peng Y (2022). CARNet: Cascade attentive RefineNet for multi-lesion segmentation of diabetic retinopathy images. Complex Intell. Syst..

[10] Darwish A (2018). Bio-inspired computing: Algorithms review, deep analysis, and the scope of applications. Future Comput. Inf. J..

[11] Wang H, Wang W, Zhou X, Sun H, Zhao J, Xiang Yu, Cui Z (2017). Firefly algorithm with neighborhood attraction. Inf. Sci..

[12] Uppamma P, Bhattacharya S (2023). Deep learning and medical image processing techniques for diabetic retinopathy: A survey of applications, challenges, and future trends. J. Healthcare Eng..

[13] Selvachandran G, Quek SG, Paramesran R, Ding W, Son LH (2023). Developments in the detection of diabetic retinopathy: a state-of-the-art review of computer-aided diagnosis and machine learning methods. Artif. Intell. Rev..

[14] Ju L, Wang X, Zhao X, Huimin Lu, Mahapatra D, Bonnington P, Ge Z (2021). Synergic adversarial label learning for grading retinal diseases via knowledge distillation and multi-task learning. IEEE J. Biomed. Health Inf..

[15] Gadekallu TR, Khare N, Bhattacharya S, Singh S, Maddikunta PKR, Srivastava G (2020). Deep neural networks to predict diabetic retinopathy. J. Ambient Intell. Human. Comput..

[16] Zhou Yi, Wang B, He X, Cui S, Shao L (2020). DR-GAN: conditional generative adversarial network for fine-grained lesion synthesis on diabetic retinopathy images. IEEE J. Biomed. Health Inf..

[17] Bilal A, Guangmin Sun Y, Li SM, Khan AQ (2021). Diabetic retinopathy detection and classification using mixed models for a disease grading database. IEEE Access.

[18] Abdelmaksoud E, El-Sappagh S, Barakat S, Abuhmed T, Elmogy M (2021). Automatic diabetic retinopathy grading system based on detecting multiple retinal lesions. IEEE Access.

[19] Farag MM, Fouad M, Abdel-Hamid AT (2022). Automatic severity classification of diabetic retinopathy based on densenet and convolutional block attention module. IEEE Access.

[20] Zhang G, Sun B, Chen Z, Gao Y, Zhang Z, Li K, Yang W (2022). Diabetic retinopathy grading by deep graph correlation network on retinal images without manual annotations. Front. Med..

[21] Chen Y, Shibao X, Long J, Xie Y (2023). DR-Net: Diabetic retinopathy detection with fusion multi-lesion segmentation and classification. Multim. Tools Appl..

[22] Aujih AB, Shapiai MI, Meriaudeau F, Tang TB (2022). EDR-Net: Lightweight deep neural network architecture for detecting referable diabetic retinopathy. IEEE Trans. Biomed. Circuits Syst..

[23] Bilal A, Sun G, Mazhar S, Imran A, Latif J (2022). A transfer learning and U-Net-based automatic detection of diabetic retinopathy from fundus images. Comput. Methods Biomech. Biomed. Eng. Imaging Visual..

[24] Abbood SH, Hamed HNA, Rahim MSM, Rehman A, Saba T, Bahaj SA (2022). Hybrid retinal image enhancement algorithm for diabetic retinopathy diagnostic using deep learning model. IEEE Access.

[25] Wang X, Mai Xu, Zhang J, Jiang L, Li L, He M, Wang N, Liu H, Wang Z (2021). Joint learning of multi-level tasks for diabetic retinopathy grading on low-resolution fundus images. IEEE J. Biomed. Health Inf..

[26] Gadekallu TR, Alazab M, Kaluri R, Maddikunta PKR, Bhattacharya S, Lakshmanna K (2021). Hand gesture classification using a novel CNN-crow search algorithm. Compl. Intell. Syst..

[27] Zang P, Gao L, Hormel TT, Wang J, You Q, Hwang TS, Jia Y (2020). DcardNet: diabetic retinopathy classification at multiple levels based on structural and angiographic optical coherence tomography. IEEE Trans. Biomed. Eng..

[28] Hua C-H, Kim K, Huynh-The T, You JI, Seung-Young Y, Le-Tien T, Bae S-H, Lee S (2020). Convolutional network with twofold feature augmentation for diabetic retinopathy recognition from multi-modal images. IEEE J. Biomed. Health Inf..

[29] Rajesh Khanna M (2023). Multi-level classification of Alzheimer disease using DCNN and ensemble deep learning techniques. Signal Image Video Process..

[30] Arunkumar R, Karthigaikumar P (2017). Multi-retinal disease classification by reduced deep learning features. Neural Comput. Appl..

[31] Huang S, Li J, Xiao Y, Shen N, Tingfa Xu (2022). RTNet: relation transformer network for diabetic retinopathy multi-lesion segmentation. IEEE Trans Med. Imag..

[32] Niu Y, Lin Gu, Zhao Y, Feng Lu (2021). Explainable diabetic retinopathy detection and retinal image generation. IEEE J. Biomed. Health Inf..

[33] Le R, Cui Y, Lu ES, Zhu Y, Garg I, Wang JC, Yifan L (2023). Prevalence of venous loops and association with retinal ischemia in diabetic retinopathy using widefield swept-source OCT angiography. Graefe's Archiv. Clin. Exp. Ophthalmol..

[34] Jena PK, Khuntia B, Palai C, Nayak M, Mishra TK, Mohanty SN (2023). A novel approach for diabetic retinopathy screening using asymmetric deep learning features. Big Data Cogn. Comp..

[35] Ayala A, Figueroa TO, Fernandes B, Cruz F (2021). Diabetic retinopathy improved detection using deep learning.". Appl. Sci..

[36] Luo X, Wang W, Yong X, Lai Z, Jin X, Zhang B, Zhang D (2023). A deep convolutional neural network for diabetic retinopathy detection via mining local and long-range dependence. CAAI Trans. Intell. Technol..

[37] Bilal A, Sun G, Mazhar S (2021). Survey on recent developments in automatic detection of diabetic retinopathy. J. Français d'Ophtalmologie.

[38] Bilal, A., Sun, G., & Mazhar, S. Diabetic retinopathy detection using weighted filters and classification using CNN. In *2021 International Conference on Intelligent Technologies* (CONIT) 1–6 (IEEE, 2021).

[39] Bilal, A., Sun, G., Mazhar, S., & Imran, A. Improved Grey Wolf optimization-based feature selection and classification using CNN for diabetic retinopathy detection. In *Evolutionary Computing and Mobile Sustainable Networks: Proceedings of ICECMSN 2021* 1–14 (Springer, Singapore, 2022).

[40] Bilal A, Zhu L, Deng A, Huihui Lu, Ning Wu (2022). AI-based automatic detection and classification of diabetic retinopathy using U-Net and deep learning. Symmetry.

[41] Chan FHY, Lam FK, Zhu H (1998). Adaptive thresholding by variational method. IEEE Trans. Image Process..

[42] Torse D, Desai V, Khanai R (2017). A review on seizure detection systems with emphasis on multi-domain feature extraction and classification using machine learning. Broad Res. Artif. Intell. Neurosci..

[43] Mirjalili S (2015). Moth-flame optimization algorithm: A novel nature-inspired heuristic paradigm. Knowledge-based Syst..

[44] Chen S, Webb GI, Liu L, Ma X (2020). A novel selective naïve Bayes algorithm. Knowledge-Based Syst..

[45] Hu Q, Daren Yu, Xie Z (2008). Neighborhood classifiers. Exp. Syst. Appl..

[46] Kwok JT-Y (1999). Moderating the outputs of support vector machine classifiers. IEEE Trans. Neural Netw..

[47] Lee K, Ahn H, Moon H, Kodell RL, Chen JJ (2013). Multinomial logistic regression ensembles. J. Biopharm. Stat..

[48] Murtagh F (1991). Multilayer perceptrons for classification and regression. Neurocomputing.

[49] Breiman L (2001). Random forests. Mach. Learn..

[50] Porwal P (2018). Fabrice IEEE Dataport 2018. Indian diabetic retinopathy image dataset. Data.

[51] Nguyen, A. H. Diaretdb1—standard diabetic retinopathy database. diaretdb1 https://www.kaggle.com/datasets/nguyenhung1903/diaretdb1-standard-diabetic-retinopathy-database (2021).

